# An assessment of the use of complementary and alternative medicine by Korean people using an adapted version of the standardized international questionnaire (I-CAM-QK): a cross-sectional study of an internet survey

**DOI:** 10.1186/s12906-018-2294-6

**Published:** 2018-08-13

**Authors:** Ju Ah Lee, Yui Sasaki, Ichiro Arai, Ho-Yeon Go, Sunju Park, Keiko Yukawa, Yun Kung Nam, Seong-Gyu Ko, Yoshiharu Motoo, Kiichiro Tsutani, Myeong Soo Lee

**Affiliations:** 1 Department of Korean Internal Medicine, College of Korean Medicine, Gachon University, Incheon, Republic of Korea; 2 Institute of Safety and Effectiveness Evaluation for Korean Medicine, Department of Preventive Medicine, College of Korean Medicine, Kyung Hee University, Seoul, Republic of Korea; 3 Department of Pharmaceutical Sciences, Nihon Pharmaceutical University, Saitama, Japan; 4 Department of Internal Medicine, College of Korean Medicine, Semyung University, Jecheon, Republic of Korea; 5 Department of Preventive Medicine, College of Korean Medicine, Daejeon University, Daejeon, Republic of Korea; 6 Department of Health Policy and Technology Assessment, National Institute of Public Health, Saitama, Japan; 7 Department of Medical Oncology, Kanazawa Medical University, Ishikawa, Japan; 8 Faculty of Health Sciences, Tokyo Ariake University of Medical and Health Sciences, Tokyo, Japan; 9 Clinical Medicine Division, Korea Institute of Oriental Medicine, 1672 Yuseong-daero, Yuseong-gu, Daejeon, 34054 Republic of Korea

**Keywords:** Complementary and alternative medicine, Korea, I-CAM-Q

## Abstract

**Background:**

In Korea, there are two types of medical doctors: one practises conventional medicine (hereafter called a physician), and the other practises traditional medicine (hereafter called a Korean medical doctor). This study aimed to compare the provision of complementary and alternative medicine (CAM) by these providers to CAM use per self-judgement in Korea.

**Methods:**

We analysed 1668 Korean people via an internet survey with the Korean adopted version of the I-CAM-Q, namely, the International Questionnaire to measure use of CAM, to understand whether respondents used CAM based either on a prescription or advice from a physician or a Korean medical doctor or on self-judgement.

**Results:**

In the previous 12 months, the proportions of respondents who were treated by a physician, who were treated by a Korean medical doctor and who were not treated by anyone were 67.9, 20.7 and 14.2%, respectively. Among the respondents who received CAM based on a prescription or advice from a physician, traditional Korean medicine practices and dietary supplements were commonly used; only a small percentage used other CAM therapies. Respondents who received CAM based on a prescription or advice from a Korean medical doctor showed similar results. Acupuncture and moxibustion, traditional Korean medicines (decoction), or cupping were more commonly used. Korean traditional medicines as over-the-counter (OTC) drugs were more commonly used by respondents who received CAM therapy based on a prescription or advice from a physician than by those who received CAM therapy based on a prescription or advice from a Korean medical doctor. A total of 74% of the responders used any CAM by self-judgement in the previous 12 months.

**Conclusions:**

For the use of CAM in Korea, in addition to the Korean traditional medical care provided by Korean medical doctors, general physicians advised people regarding Korean traditional medical care and dietary supplements.

**Electronic supplementary material:**

The online version of this article (10.1186/s12906-018-2294-6) contains supplementary material, which is available to authorized users.

## Background

Traditional medicine is classified as complementary and alternative medicine (CAM) in many countries. However, in East Asian countries, such as Korea, Japan, and China, traditional medicine is now used not as CAM but as official medicine; nevertheless, in previous surveys of CAM use in these countries, almost all studies classified traditional medicine as CAM [1–17].

The reports on the utilization of CAM in South Korea (hereafter Korea) through 2011 have been reviewed by Kim SG et al. [1]. There have also been several other reports since 2011 [2–11]. However, most studies either report on the utilization of CAM in patients with specific diseases (e.g., cancer) or provide an indirect report. According to a direct survey on the utilization of CAM throughout Korea (performed by Ock SM et al. [12]) in 2006, 74.8% of Koreans had used some type of CAM therapy in the past 12 months.

The results of the survey on the utilization of CAM may be significantly influenced by the manner of questioning; moreover, the results of the surveys may vary greatly depending on the contents of the questionnaires, even if the surveys are conducted in the same country [18]. Therefore, in an international group of CAM researchers, a standardized questionnaire, the International Questionnaire to measure use of Complementary and Alternative Medicine (I-CAM-Q), was developed to reduce questionnaire-related bias and to conduct an international comparison of the utilization of CAM [19]. The use of the I-CAM-Q was originally limited; then, several surveys on the utilization of CAM were conducted using this questionnaire in several countries [20–30]. As the type and circumstances of CAM may vary greatly according to differences in the cultural backgrounds of each country, an adapted version of the I-CAM-Q may be developed before implementation in a survey [23, 27].

This paper describes the results of a survey in Korea on the use of CAM that includes Korean traditional medicine. This survey was carried out using the I-CAM-Q for the first time and included CAM providers’ viewpoints.

## Methods

### Development of draft questionnaire

Using the original I-CAM-Q for a survey of CAM without adapting it to the environment of Korea makes the I-CAM-Q not suitable. Therefore, we first developed the adapted version of the I-CAM-Q for Korea by referring to the I-CAM-QJ, the adapted version of the I-CAM-Q for Japan [31]. We referred to the I-CAM-QJ because Japan uses traditional medicine derived from ancient China as the official medicine, and the cultural background for CAM is similar to that of Korea. To develop the I-CAM-QK (Korean version of I-CAM-Q; Additional file 1), the most important revision from the original I-CAM-Q was the addition of “Korean medical doctor” as a healthcare provider. In Korea, there are two types of medical doctors’ licences: one is for conventional medicine, and the other is for traditional medicine. Korean medical doctors can prescribe traditional Korean medicines and practice acupuncture and moxibustion and provide other traditional remedies. In addition, in the option list, CAM treatments that are used frequently, such as “Cupping”, in Korea were added. The herbal medicines listed in the options were changed to the most commonly used ones in Korea by referring to the documents of the National Health Insurance Service of Korea [32]. Additionally, we changed the options in dietary supplements by referring to the documents of the Ministry of Food and Drug Safety of Korea [33].

### Validation and revision of questionnaire by a pilot interview survey

Using the draft questionnaire according to the abovementioned policies, an interview survey was conducted involving 30 Korean people at Daejeon University and Semyung University (Semyung University, IRB No. 1608–07) in Korea between September 2016 and December 2016. Based on the results of this survey, we slightly revised some terms of the questionnaires to prevent misunderstanding by the respondents.

### Internet survey

Between February 24 and March 3, 2017, a survey company in Japan (ANTERIO Inc.) conducted an internet survey using the I-CAM-QK.

The survey method was as follows. A tie-ups company of Anterio has over 500,000 panels (willing partners for internet interviews) in Korea. For each generation, namely, people in their twenties, thirties, forties, and fifties/sixties, approximately 200 samples of both males and females were needed (200 × 2 × 4 = 1600). We excluded people who were over 69 years old because most of them in Korea might not use the internet and were not registered in the panel. We also excluded people who were under 20 years old because they might answer the questions incorrectly and were not registered in our panel. We collected the same number of answers from each generation/sex for comparisons to surveys in other countries that we plan to perform.

The survey implementation date was February 24, 2017. The database of internet addresses included those of the general population. No specific group of people was over-represented. There was no stratification such as according to the region of the country. The internet survey began by requesting 200 samples as the target number for each sex and generation group via e-mail. The questionnaire was e-mailed again to groups that had not been reached. People working in any of the following areas were excluded from the survey: medicine, media, advertising and market research. We excluded these people because they may have presumed the purpose of the survey and not answered honestly. The average time to answer was 28 min (median 7 min).

This internet survey was performed after obtaining the approval of the ethics committee of Nihon Pharmaceutical University (approved number: 28–05) and of the institutional review board (IRB) of the Second Affiliated Korean Medical Hospital in Chungju, Semyung University (Semyung University IRB No. 1702–01). Before initiation, this survey was registered in the University Hospital Medical Information Network in Japan (UMIN000026399).

## Results

### Demographics

Table 1 shows the attributes of the respondents. A total of 1668 people were enrolled as respondents, who were equally distributed in each age group and sex group. For their current health condition, approximately 15% of respondents indicated that they were in ‘bad’ and ‘very bad’ health, while the rest indicated that they were at least in ‘acceptable’ health. Regarding educational background, the percentage of respondents who graduated from a university was approximately 60%. In terms of long-term disease/disorders, 32.3% of the respondents indicated that they had at least one; hypertension, gastrointestinal disease, dental disease and skin disease were commonly found in high proportions (> 20% for each).

**Table 1:** Demographics

	Number	%
Total Number (Male/Female)	1668	
Male / Female	831 / 837	49.8 / 50.2
Age (Male/Female)		
20’s Male / 20’s Female	205 / 210	12.3 / 12.6
30’s Male / 30’s Female	210 / 217	12.6 / 13.0
40’s Male / 40’s Female	209 / 210	12.6 / 12.6
50~ 60’s Male / 50~ 60’s Female	207 / 200	12.4 / 12.0
Profession		
Agriculture, fisheries, forestry, mining	16	1.0
Productions	242	14.6
Retails	86	5.2
Civil engineering and construction, Real Estate, Building Services, Transportation, Warehouse Logistics related	181	10.9
Communication industry, Software, Information processing, Other information service	105	6.3
Electricity, Gas, Heat supply Water supply	26	1.6
Finance, Insurance	39	2.3
Food, Accommodation, Travel services	26	1.6
Education, Learning support	155	9.3
Welfare	32	1.9
Housewife	281	16.8
Student	158	9.5
Unemployed	123	7.4
Others	198	11.8
Final Education		
Middle school	15	0.9
High school	321	19.2
Special college	226	13.5
University	965	57.9
Graduate school	134	8.0
Others	7	0.4
Health condition		
Very good	141	8.5
Good	562	33.7
Acceptable	708	42.4
Bad	241	14.4
Long-term diseases and disorders		
Yes	539	32.3
Hypertension	140	26.0
Stroke (cerebral hemorrhage, cerebral infarction, etc.)	12	2.2
Heart disease	15	2.8
Diabetes	54	10.0
Dyslipidemia (hyperlipidemia)	54	10.0
Respiratory illness	65	12.1
Diseases of the gastrointestinal tract (gastrointestinal, liver, gall bladder, pancreas, etc.)	139	25.8
Kidney and urological diseases	43	8.0
Musculoskeletal diseases (osteoporosis, arthropathy, back pain, etc.)	103	19.1
Trauma (falls, fractures, etc.)	13	2.4
Cancer (including blood cancer and sarcoma)	12	2.2
Blood disease (other than tumor)	14	2.6
Immune disease (such as collagen disease)	18	3.3
Mental disorders such as depression / dementia	54	10.0
Nose disease	94	17.4
Eye disease	80	14.8
Ear disease	39	7.2
Skin disease	114	21.2
Tooth disease	123	22.8
Others	51	9.5
No	1129	67.7
Subscribing to private medical insurance		
Subscribe	1109	66.5
Not subscribe	559	33.5

### Healthcare providers

As shown in Table 2, 67.9% of respondents indicated that they received medical care/health services from physicians in the last 12 months. On average, participants visited 2.2 different health providers in the last 12 months. In contrast, 20.7% of respondents indicated that they received medical care/health services from Korean medical doctors in the last 12 months, which was equivalent to one-third of the respondents who received the services from physicians. Regarding services from providers other than physicians, the respondents received services from dentists, pharmacists and nurses (35.9, 43.5, and 43.5%, respectively). In terms of the services from CAM providers other than Korean medical doctors, the percentage of respondents who received services from massage practitioners or acupressure therapists was relatively high, at 9.7%. However, the percentage of respondents who received CAM therapies from other providers was low, at < 4%.

**Table 2:** Visiting health care providers

Health care providers	Visited^a^ (%)	Motivation^a^ (%)				Helpfulness^a^ (%) (Very and somewhat)	Number of visit^b^
		Acute illness^c^	Long-term illness^d^	Improvement of well-being	Others		
Physicians	67.9	35.1	30.0	27.7	7.2	94.8	2.6
Korean medical doctor	20.7	26.7	29.6	38.3	5.5	89.6	3.0
Dentist	35.9	23.1	22.2	46.5	8.2	97.0	1.7
Pharmacist	43.5	42.0	20.7	33.1	4.3	93.2	3.0
Nurse	22.4	41.4	19.8	31.8	7.0	93.6	2.9
Maternity nurse	0.1	0.0	0.0	0.0	100.0	100.0	1.0
Massage practitioner / Acupressure therapist	9.7	6.8	14.8	75.3	3.1	89.5	2.8
Acupuncturist / Moxibustionist	2.0	21.2	42.4	36.4	0.0	90.9	2.9
Judo therapist (Bonesetter)	0.3	40.0	40.0	20.0	0.0	100.0	2.2
Nutritionist	1.6	3.7	0.0	92.6	3.7	77.8	4.9
Yoga instructor	3.6	0.0	6.7	90.0	3.3	88.4	11.5
Chiropractor	1.4	20.8	37.5	41.7	0.0	83.4	2.2
Manual therapist	3.7	24.6	34.4	39.3	1.6	88.6	2.8
Aromatherapist / Herb therapist	3.2	11.3	7.5	75.5	5.7	84.9	2.3
Spiritual therapist	0.8	0.0	61.5	38.5	0.0	92.3	1.6
Homeopathy therapist	0.6	30.0	40.0	30.0	0.0	90.0	3.0
Others	0.8	0.1	0.2	0.3	0.1	80.0	2.4
Not received medical / health services	14.2	NA	NA	NA	NA	NA	1.0

Number of respondents: 1668

*NA* Not Applicable

^a^ in the last 12 months, ^b^ in the last 3 months, ^c^ lasted less than 1 month, ^d^ lasted more than 1 month

For the reasons provided by respondents for seeing a Korean medical doctor, the percentage of those who reported improved well-being was high, while the percentage of those who reported acute illness was low. The percentages of respondents who were satisfied with the services provided by physicians and Korean medical doctors were 94.8 and 89.5%, respectively.

### CAM treatments received from physicians

The respondents who received medical services from physicians (1132 respondents) were asked to answer a question regarding ‘medical/healthcare services that were used based on the advice of physicians in the last 12 months’ (Table 3). The most common CAM used by these respondents was over-the-counter (OTC) traditional Korean medicine, which was used by 26.7% of the 1132 respondents, equivalent to 18.1% of all 1668 respondents. This was followed by dietary supplementation (18.5% of 1132 respondents; 12.6% of all respondents), acupuncture and moxibustion (15.9%; 10.8%), manufactured traditional Korean medicine for prescription (11.4%; 7.7%) and traditional Korean medicines (decoction) (9.0%; 6.1%). Of the respondents who received traditional Korean medicines, the number of respondents who used OTC traditional Korean medicine was highest, whereas the number of respondents who used decoctions was lowest.

**Table 3:** CAM treatments received by practice or advices of physicians

CAM treatments	Received^b^ (%)		Motivation^b^ (%)				Helpfulness^b^ (%) (Very and somewhat)	Number of visit^c^
	among the visitors to physicians (1132)	among total respondents^a^ (1668)	Acute illness^d^	Long-term illness^e^	Improvement of well-being	Others		
Acupuncture and moxibustion	15.9	10.8	28.9	33.3	35.6	2.2	88.9	4.6
Massage	8.1	5.5	13.0	22.8	60.9	3.3	85.9	3.7
Bone-setting	1.0	0.7	45.5	36.4	9.1	9.1	81.9	2.9
Bodywork	6.2	4.2	25.7	34.3	37.1	2.9	81.5	3.2
Chiropractic	2.4	1.6	29.6	33.3	37.0	0.0	92.6	2.1
Cupping	6.1	4.1	33.3	21.7	39.1	5.8	85.5	3.0
Diet therapy	8.0	5.4	9.9	33.0	57.1	0.0	83.5	4.1
Starvation diet	1.3	0.9	26.7	20.0	46.7	6.7	73.4	2.3
Dietary supplement	18.5	12.6	3.8	21.5	73.7	1.0	80.4	12.4
Herb therapy	1.2	0.8	7.1	28.6	64.3	0.0	71.4	2.0
Aromatherapy	2.4	1.6	11.1	18.5	63.0	7.4	96.3	4.1
Hyperthermia	7.6	5.2	29.1	32.6	34.9	3.5	89.5	4.1
Magnet therapy	1.8	1.2	30.0	25.0	45.0	0.0	95.0	3.4
Spa therapy	3.4	2.3	5.1	17.9	76.9	0.0	89.7	3.3
Music therapy	1.2	0.8	14.3	35.7	50.0	0.0	78.6	2.4
Forest therapy	1.0	0.7	0.0	18.2	81.8	0.0	72.8	1.5
Homeopathy	0.7	0.5	50.0	12.5	37.5	0.0	87.5	1.4
Ayurveda	0.3	0.2	33.3	33.3	33.3	0.0	100.0	1.3
Yoga	3.4	2.3	5.3	7.9	81.6	5.3	81.6	10.0
Qigong	1.1	0.7	15.4	38.5	46.2	0.0	92.3	6.2
Traditional Korean medicines (decoction)	9.0	6.1	24.5	31.4	42.2	2.0	90.2	1.8
Manufactured traditional Korean medicines for prescription	11.4	7.7	30.2	26.4	41.1	2.3	88.3	2.1
OTC traditional Korean medicines	26.7	18.1	36.4	24.2	36.4	3.0	84.8	2.5
Spiritual therapy	1.8	1.2	5.0	50.0	45.0	0.0	80.0	2.9
Other	35.6	24.2	16.3	42.9	32.7	8.2	85.8	6.3

*CAM* Complementary and Alternative Medicine, *NA* Not Applicable

Number of parent population except ^(a)^ is the visitors to physicians (1132)

^b^ in the last 12 months, ^c^ in the last 3 months, ^d^ lasted less than 1 month, ^e^ lasted more than 1 month

### CAM treatments received from Korean medical doctors

The respondents who received services from Korean medical doctors (345 respondents) were asked to answer a question regarding ‘medical/healthcare services that were used based on the advice of Korean medical doctors in the last 12 months’ (Table 4). The CAM used most commonly, by 69.0% of the 345 respondents, which was equivalent to 14.3% of all 1668 respondents, was acupuncture and moxibustion. This was followed by traditional Korean medicine (decoction) (36.2% of 345 respondents; 7.5% of all respondents) and cupping (32.5%; 6.7%). Of the respondents who received traditional Korean medicine, the number of respondents who used decoction was highest, while the number of those who used OTC traditional Korean medicine was lowest. The helpfulness of these CAM treatments was largely reported as similar to the results regarding the CAM therapies that the respondents used based on a prescription or advice from a physician. For medical care/health services that the respondents used based on a prescription or advice from a Korean medical doctor, the availability of CAM other than traditional Korean medicine was low.

**Table 4:** CAM treatments received by practice or advices of Korean medical doctors

CAM treatments	Received^b^ (%)		Motivation^b^ (%)				Helpfulness^b^ (%) (Very and somewhat)	Number of visit^c^
	among the visitors to Korean medical doctors (345)	among total respondents^a^ (1668)	Acute illness^d^	Long-term illness^e^	Improvement of well-being	Others		
Acupuncture and moxibustion	69.0	14.3	33.6	33.6	29.8	2.9	89.1	4.6
Massage	11.6	2.4	20.0	27.5	50.0	2.5	95.0	2.7
Bone-setting	2.0	0.4	42.9	14.3	42.9	0.0	100.0	5.3
Bodywork	6.7	1.4	43.5	34.8	21.7	0.0	95.6	3.3
Chiropractic	7.5	1.6	30.8	50.0	11.5	7.7	88.5	2.3
Cupping	32.5	6.7	29.5	31.3	33.0	6.3	84.9	3.8
Diet therapy	7.0	1.4	8.3	29.2	62.5	0.0	91.6	8.2
Starvation diet	1.7	0.4	33.3	16.7	33.3	16.7	66.6	3.0
Dietary supplement	8.4	1.7	3.4	17.2	79.3	0.0	68.9	12.4
Herb therapy	1.7	0.4	0.0	33.3	66.7	0.0	83.4	2.0
Aromatherapy	2.9	0.6	0.0	20.0	80.0	0.0	80.0	3.0
Hyperthermia	15.4	3.2	24.5	39.6	30.2	5.7	90.6	4.2
Magnet therapy	4.3	0.9	26.7	46.7	26.7	0.0	93.3	5.8
Spa therapy	3.5	0.7	0.0	50.0	50.0	0.0	100.0	2.2
Music therapy	2.0	0.4	0.0	42.9	57.1	0.0	100.0	4.1
Forest therapy	2.0	0.4	28.6	28.6	42.9	0.0	100.0	2.1
Homeopathy	0.3	0.1	0.0	100.0	0.0	0.0	100.0	0.0
Ayurveda	0.9	0.2	0.0	33.3	66.7	0.0	66.6	2.3
Yoga	2.0	0.4	14.3	14.3	71.4	0.0	85.7	3.3
Qigong	2.0	0.4	28.6	57.1	14.3	0.0	71.4	7.1
Traditional Korean medicines (decoction)	36.2	7.5	19.2	30.4	48.8	1.6	87.2	2.8
Manufactured traditional Korean medicines for prescription	19.7	4.1	25.0	33.8	35.3	5.9	85.3	3.1
OTC traditional Korean medicines	10.4	2.2	19.4	36.1	44.4	0.0	91.7	5.4
Spiritual therapy	1.4	0.3	20.0	80.0	0.0	0.0	80.0	3.8
Other	4.7	1.0	50.0	50.0	0.0	0.0	100.0	1.0

*CAM* Complementary and Alternative Medicine, *NA* Not Applicable

Number of parent population except ^(a)^ is the visitors to Korean medical doctors (345)

^b^ in the last 12 months, ^c^ in the last 3 months, ^d^ lasted less than 1 month, ^e^ lasted more than 1 month

### Self-help practices

All 1668 respondents were asked to answer a question regarding whether they engaged in ‘self-help practices in the last 12 months’ (Table 5). The respondents who did not engage in such practices in the last 12 months made up 26.3% of the respondents; the rest engaged in some type of self-help practice. The most common self-help practice that the respondents reported was walking (54.9%). The least common self-help practices that at least 10% of the respondents reported were yoga, use of an electric massage machine and spa therapy (13.2, 10.1 and 10.0%, respectively). The proportion of respondents who engaged in self-help practices to improve their well-being was higher than that of the respondents who used CAM per a prescription or advice from physicians/Korean medical doctors.

**Table 5:** Self-help practices

CAM treatments	Used^a^ (%)	Motivation^a^ (%)				Helpfulness^a^ (%) (Very and somewhat)	number of times practice^b^
		Acute illness^c^	Long-term illness^d^	Improvement of well-being	Others		
Meditation	9.6	3.1	10.6	85.0	1.3	88.8	12.7
Yoga	13.2	3.6	10.9	82.3	3.2	93.2	15.2
Qigong	1.6	7.4	25.9	63.0	3.7	96.3	14.0
Tai Chi	0.7	25.0	25.0	50.0	0.0	100.0	12.0
Relaxation techniques	2.5	12.2	31.7	56.1	0.0	85.4	4.9
Music therapy	4.2	1.4	18.6	71.4	8.6	94.3	15.5
Picture therapy	1.1	5.6	22.2	50.0	22.2	88.9	4.3
Attend traditional healing ceremony	1.1	5.6	22.2	72.2	0.0	94.5	1.9
Praying for own health	5.9	5.1	10.2	82.7	2.0	85.8	25.7
Electric massage machine	10.1	14.8	16.6	66.9	1.8	82.9	11.0
Other health appliances	7.1	5.0	16.8	78.2	0.0	89.9	22.6
Walking	54.9	1.5	5.9	91.4	1.2	88.3	27.9
Forest therapy	4.6	1.3	13.0	85.7	0.0	85.7	4.2
Aromatherapy	5.5	7.7	12.1	76.9	3.3	81.3	8.0
Hyperthermia	7.1	16.0	26.9	56.3	0.8	89.9	9.4
Magnet therapy	2.7	15.6	35.6	48.9	0.0	86.7	9.6
Spa therapy	10.0	6.0	11.4	81.3	1.2	86.2	5.2
Bath additive	8.3	3.6	8.7	81.9	5.8	73.9	6.2
Others	1.2	0.0	15.0	80.0	5.0	100.0	35.0
Not received	26.3	NA	NA	NA	NA	NA	NA

Number of respondents: 1668

*CAM* Complementary and Alternative Medicine, *NA* Not Applicable

^a^ in the last 12 months, ^b^ in the last 3 months, ^c^ lasted less than 1 month, ^d^ lasted more than 1 month

### Use of dietary supplements

All 1668 respondents were asked to answer a question regarding which dietary supplements they used (Table 6). The most common dietary supplement that the respondents used was vitamin C (35.1%), followed by red ginseng (30.8%), omega-3 fatty acids (29.3%) and multivitamins (22.0%). These were all used to improve well-being. However, the percentage of respondents who indicated that it was helpful was < 80% (except in cases of dietary supplements, which few respondents used).

**Table 6:** Use of dietary supplements

Dietary supplements	Used^a^ (%)	Use now (%)	Motivation^a^ (%)				Helpfulness^a^ (%) (Very and somewhat)
			Acute Illness^b^	Long-term Illness^c^	Improvement of well-being	Others	
Vitamin A	8.9	5.2	4.7	8.1	86.5	0.7	76.4
Vitamin B_1_	5.0	2.8	4.8	14.5	80.7	0.0	78.4
Vitamin B_2_	5.1	3.0	7.1	8.2	84.7	0.0	78.8
Vitamin B_6_	3.3	1.9	0.0	9.1	90.9	0.0	74.6
Vitamin B_12_	3.9	1.9	9.2	9.2	81.5	0.0	70.8
Vitamin C	35.1	27.1	1.4	5.0	92.3	1.4	74.0
Vitamin D	15.9	11.3	1.9	12.4	83.8	1.9	72.2
Vitamin E	3.8	2.0	3.1	6.3	90.6	0.0	78.1
Vitamin K	1.9	0.7	6.5	3.2	90.3	0.0	70.9
Multiple vitamin	22.0	18.8	0.5	5.2	92.9	1.4	73.6
Pantothenic acid	0.6	0.4	10.0	0.0	90.0	0.0	90.0
Biotin	1.9	1.1	3.1	18.8	78.1	0.0	59.4
Niacin	0.9	0.5	13.3	13.3	73.3	0.0	60.0
Folic acid	6.7	3.9	2.7	9.9	72.1	15.3	74.8
Iron	5.7	3.5	8.4	16.8	69.5	5.3	78.9
Calcium	11.9	7.6	2.0	11.1	85.4	1.5	68.7
Copper	0.4	0.2	0.0	0.0	100.0	0.0	50.0
Zinc	4.9	2.8	3.7	7.4	88.9	0.0	61.7
Magnesium	9.5	5.6	9.4	5.7	83.6	1.3	63.5
Potassium	4.1	2.0	7.4	11.8	80.9	0.0	70.6
Multi-mineral	6.1	3.7	2.0	6.9	91.1	0.0	67.3
Glucosamine	5.3	3.4	2.2	12.4	84.3	1.1	69.7
Chondroitin	0.2	0.1	0.0	0.0	100.0	0.0	100.0
Saw palmetto	0.2	0.2	0.0	75.0	25.0	0.0	75.0
Green juice	3.3	1.9	7.3	14.5	78.2	0.0	80.0
Collagen	3.5	1.7	6.8	15.3	78.0	0.0	67.8
Placenta	0.4	0.2	16.7	50.0	33.3	0.0	50.0
Blueberry	8.0	4.7	1.5	3.0	92.5	3.0	59.4
Red ginseng	30.8	21.5	1.0	5.1	91.8	2.1	75.7
Ginseng	4.6	2.8	1.3	3.9	94.8	0.0	77.9
Omega-3 fatty acid	29.2	21.0	1.2	9.9	87.7	1.2	67.4
Probiotic	15.8	11.2	3.0	13.3	83.3	0.4	78.1
Others	3.0	2.6	0.0	6.0	90.0	4.0	78.0
Not used	18.3	25.0	NA	NA	NA	NA	NA

Number of respondents: 1668

*NA* Not Applicable

^a^ in the last 12 months, ^b^ lasted less than 1 month, ^c^ lasted more than 1 month

The respondents (1326) who indicated that they used dietary supplements were asked about their purchasing methods; pharmacy was most common (47.5%), followed by internet shopping (45.9%), drugstore (12.8%), supermarket (8.2%) and mail order (4.8%), which suggested that half or more of the respondents bought their dietary supplements at actual stores (table not shown).

### Use of traditional Korean medicine

All 1668 respondents were asked to answer a question regarding the type of traditional Korean medicine that they used in the last 12 months. The most common type was OTC manufactured Korean medicinal products (32.1%), followed by prescribed manufactured Korean medicinal products (18.5%) and decoctions dispensed by a Korean medical doctor (18.2%) (data not shown).

The 831 respondents who indicated that they had used traditional Korean medicine in the last 12 months were asked to recall the names of the specific medications (Table 7). The most common type that they used was galguentang (kakkonto in Japanese, ge-gen-tang in Chinese; 30.0%); the percentage of each other prescription that was used was < 6%.

**Table 7:** Use of Korean medicines

Korean medicines	Used^a^ (%)	Use now (%)	Motivation^a^ (%)				Helpfulness^a^ (%) (Very and somewhat)
			Acute illness^b^	Long-term Illness^c^	Improvement of well-being	Others	
Ojeogsan	5.5	2.0	19.6	23.9	56.5	0.0	78.2
Gunghatang	2.4	1.3	15.0	50.0	35.0	0.0	95.0
Ijintang	3.1	1.9	19.2	34.6	46.2	0.0	76.9
Gumiganghwaltang	3.0	1.9	8.0	32.0	60.0	0.0	88.0
Pyeongwisan	3.5	1.7	24.1	34.5	41.4	0.0	96.5
Hyangsapyeongwisan	3.5	1.9	24.1	34.5	41.4	0.0	89.6
Bojungikgitang	5.7	4.2	8.5	34.0	57.4	0.0	87.2
Socheongryongtang	4.2	2.5	14.3	22.9	60.0	2.9	82.9
Galguentang	30.0	15.5	36.5	14.1	47.8	1.6	82.4
Samsoeum	3.1	1.4	23.1	19.2	57.7	0.0	88.4
Others	7.6	3.2	14.3	11.1	63.5	11.1	80.9
Not know / Not remember	47.4	17.6	NA	NA	NA	NA	NA
Not used	0.0	51.7	NA	NA	NA	NA	NA

Number of respondents: 831

*NA* Not Applicable

^a^ in the last 12 months, ^b^ lasted less than 1 month, ^c^ lasted more than 1 month

## Discussion

The I-CAM-Q used for our survey was not a questionnaire to gain factual information on various kinds of CAM but a questionnaire regarding utilization, motivation and helpfulness by distinguishing ‘CAM provided by whom’.

In Korea, unlike Western countries and Japan, a licence to practise traditional medicine, as well as a general physician’s licence, is authorised; thus, observing how both types of licensed physician are involved in the use of CAM is interesting. Generally, the term ‘integrative medicine’ is used to combine medical care with CAM. However, in Korea, there may be multiple integrations, including an integration of ‘CAM, Western medicine and traditional medicine’, which is provided by physicians, or an integration of ‘traditional medicine and other CAMs’, which is provided by Korean medical doctors.

When Korean people receive treatment by physicians, they determine whether to consult a physician or a Korean medical doctor according to their disease and symptoms based on their own judgement. In this survey, 67.9% of respondents consulted a physician, and 20.7% consulted a Korean medical doctor in the last 12 months (Table 2). Considering that institutions for Korean medical care made up 21.9% of all medical institutions in Korea in 2015 [34], these results were consistent with the proportion of respondents who consulted Korean medical doctors. The most common reason the respondents consulted a physician was acute illness, while the most common reason they consulted a Korean medical doctor was to improve well-being, which suggested that respondents used both types of specialists properly.

The CAM therapies that respondents used based on a prescription or advice from a physician or Korean medical doctor were traditional Korean medicine and dietary supplements (Tables 3 and 4). Notably, the availability of other CAM therapies was lower. One can expect Korean medical doctors to provide prescriptions and advice regarding traditional Korean medicine. However, physicians were shown to also provide prescriptions and advice regarding traditional Korean medicine. In Korea, as physicians cannot prescribe traditional Korean medicine directly, the previous understanding was that physicians would advise people to receive traditional Korean medical care.

By expressing CAM therapy based on a prescription or advice from a physician or Korean medical doctor as the percentage of all 1668 respondents, comparisons could be made regarding whether CAM therapy was used based on a prescription or advice from a physician or a Korean medical doctor (Tables 3 and 4). The respondents who received acupuncture and moxibustion from physicians and/or Korean medical doctors made up 10.8 and 14.3% of all respondents, respectively, which indicated that the number of respondents who received a prescription or advice from a Korean medical doctor was higher. Similarly, 4.1 and 6.7% of all respondents received cupping from a physician or Korean medical doctor, respectively; 6.1 and 7.5% received traditional Korean medicine (decoction) based on a prescription or advice from a physician or Korean medical doctor, respectively, which also indicated that the number of respondents who received a prescription or advice from a Korean medical doctor was higher. Although both manufactured traditional Korean medicines for prescription and OTC Korean medicines are classified as traditional Korean traditional medical care, the percentage of respondents who received them from physicians was higher than that of respondents who received them from Korean medical doctors; 7.7 and 4.1% of all respondents received manufactured traditional Korean medicine for a prescription from a physician and a Korean medical doctor, respectively, and 18.1 and 2.2% of all respondents received OTC Korean medicines from a physician and a Korean medical doctor, respectively.

Notably, OTC Korean medicine was used predominately based on the advice from physicians. Thus, Korean medical doctors originally provided direct prescriptions and procedures for legal traditional Korean medicine, but they did not provide advice for OTC Korean medicine that they did not prescribe themselves, as that role was filled by general physicians. By calculating the proportion of dietary supplement use across all respondents in the same manner, we observed that Korean medical doctors were largely uninvolved in the dietary supplement regimens of the respondents: 12.6 and 1.7% of respondents used supplements based on a prescription or advice from a physician and Korean medical doctor, respectively. The proportion of respondents who used all CAM therapies, other than acupuncture and moxibustion, cupping or traditional Korean medicines (decoction), based on a prescription or advice from a physician was higher than the proportion of respondents who used these CAM therapies based on a prescription or advice from a Korean medical doctor. However, because the absolute availability of CAM therapies other than Korean medicine and dietary supplements was lower, assessing the data precisely was difficult.

This study has several limitations. The study involved an internet survey and excluded people who were over 69 years old because they were not accustomed to internet use. We also excluded people who were under 20 years old because they might answer the questions incorrectly and were not registered in our panel. Thus, this internet survey does not completely reflect the demographics of the Korean population. Because we understand the difficulty in reflecting the Korean demographics in this study, we changed the primary aim to a comparison among East Asian countries, and we are now surveying the same generations in these countries. In the future, this study will be useful with this outlook. For the survey to reflect genuine demographics, other methods, such as direct interviews, will be required. However, the direct interview may have many other difficulties, such as variations in the personality of the interviewer. Therefore, an actual conditional survey of CAM should be conducted from many standpoints.

## Conclusions

For the use of CAM in Korea, in addition to the traditional Korean medical care that is provided by Korean medical doctors, general physicians advised people regarding traditional Korean medical care and dietary supplements.

## Additional file

Additional file 1: English version of the I-CAM-QK. (DOCX 132 kb)

## Abbreviations

CAM: Complementary and alternative medicine
I-CAM-Q: International Questionnaire to measure use of Complementary and Alternative Medicine
OTC: Over-the-counter
