# Supplementary material for: An assessment of the use of complementary and alternative medicine by Korean people using an adapted version of the standardized international questionnaire (I-CAM-QK): a cross-sectional study of an internet survey
Source: BMC Complement Altern Med. 2018 Aug 13;18:238. doi: 10.1186/s12906-018-2294-6 (PMC6090824; doi:10.1186/s12906-018-2294-6)
Supplement: Supplementary file 1 — English version of the I-CAM-QK. (DOCX 132 kb) [file 12906_2018_2294_MOESM1_ESM.docx]

**A questionnaire in relation to medical care / health services.**

**(I-CAM-QＫ)**

The purpose of this survey is to gather more detail around how people are using our medical care and health services.

We ask that those people who are not medical professionals (hospital and clinic employees, pharmacists and so on),

involved in advertising or the media, or the market research industry please participate in this survey.

This survey involves questions about your own personal health, and also the hospital itself.

This questionnaire is composed from five questions:

0) Questions about your own

1) Health care providers

2) Medical care / health services received from physicians

3) Medical care / health services received from Korean Medical doctors

4) Dietary Supplements

5) Self-help practices


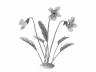
The results of this survey, once analysed, will be presented at a medical conference. In addition, once analysis is complete, the findings will be presented in a medical journal. Although the findings will be presented in various ways, under no circumstances will any personal information be included in any presentation or article.

The data taken from this survey will only be used for the purposes of this survey, and will not

be used for any other purpose. Participation in this questionnaire is purely optional. In the event

that you do not wish to participate, you will not be disadvantaged in any way for not taking part.

You agree that your answers can be used for the purposes of research and the publication thereof.

Thank you for your cooperation.

1

**Question 0. Questions about your own**

**Date**（ MM/DD/YYYY ）

Please check / fill out this questionnaire.

Q0. What is your occupation? （Check only one）

1. Agriculture, fisheries, forestry, mining 2. Civil engineering and construction 3. Real Estate, Building Services

4. Manufacture of beverages and foods 5. Manufacture of cosmetic and daily goods 6. Manufacture of electrical products

7. Manufacture and sales of automobiles and motorbikes 8. Manufacture and sales of automobiles and motorbikes

9. Other manufacturing industry 10. Department store 11. Convenience store 12. Other retail

13. Transportation, Warehouse Logistics related 14. Electricity, Gas, Heat supply Water supply 15. Communication industry

16. Software, Information processing, Other information service 17. Mass media, Advertisement Newspaper Broadcasting

18．Market research, Think-tank 19．Finance, Insurance 20. Food, Accommodation, Travel services 21. Education, Learning support

22. Medical 23. Welfare 24. Other civil servants 25. Other civil servants 26. Student 27. Unemployed 28. Unemployed

29. Others（other specify：　　　　　　　　　　　　　　　　　　　　　　　　）

Q1. What is your date of birth?　**（ MM/DD/YYYY ）**

Q2. Are you male or female? **(1. Male　２. Female)**

Q3. What is your final academic background?（Check only one）

**（1. Middle school 2. High school 3. Special college 4. University 5. Graduate school**

**6. Others (other specify：　　　　　　　　　　　　　　　　　　　　　　　　　)　）**

Q4. How is your general health condition? （Check only one）

**（　1. Very good 2. Good 3. Acceptable 4. Bad 5. Very Bad　）**

Q5. Do you have a longtime disease or disorder? (‘a longtime’ means that you have the symptom more than one month)

**（　１. Yes　２. No　）**

Q6. If you YES, which diseases？（Check all that apply）

1. Hypertension 2. Stroke (cerebral hemorrhage, cerebral infarction, etc.) 3. Heart disease 4. Diabetes 5. Dyslipidemia (hyperlipidemia)

6. Respiratory illness 7. Diseases of the gastrointestinal tract (gastrointestinal, liver, gall bladder, pancreas, etc.)

8. Kidney and urological diseases 9. Musculoskeletal diseases (osteoporosis, arthropathy, back pain, etc.) 10. Trauma (falls, fractures, etc.) 11. Cancer (including blood cancer and sarcoma) 12. Blood disease (other than tumor)　13. Immune disease (such as collagen disease)

14. Mental disorders such as depression / dementia 15. Nose disease 16. Eye disease 17. Ear disease 18．Skin disease

19．Tooth disease 20. Others（other specify：　　　　　　　　　　　　　　　　　　　　　　　　）

Q7． Do you have a private medical insurance？**（　１. Yes　２. No　）**

**Question １. Health care providers**

| Have you seen any of the following providers **in the last 12 months**? | | Number of times you saw this provider **in the last 3 months**? | Please indicate the **main** reason you **last** saw the provider.  (Check only one) | | | | | How helpful was it for you to see this provider? (Check only one) | | | |
| --- | --- | --- | --- | --- | --- | --- | --- | --- | --- | --- | --- |
|  |  |  | 1 | 2 | 3 | 4 |  | 1 | 2 | 3 | 4 |
|  |  |  | For an acute illness / condition, one that lasted less than one month | To treat a long-term health condition(one that lasted more than one month) or its symptoms | To improve well-being | Other | (Please specify the other reason) | Very | Somewhat | Not at all | Don’t know |
| **Physician** | Yes・No | times | 1 | 2 | 3 | 4 |  | 1 | 2 | 3 | 4 |
| **Korean Medical Doctor** | Yes・No | times | 1 | 2 | 3 | 4 |  | 1 | 2 | 3 | 4 |
| **Dentist** | Yes・No | times | 1 | 2 | 3 | 4 |  | 1 | 2 | 3 | 4 |
| **Pharmacist** | Yes・No | times | 1 | 2 | 3 | 4 |  | 1 | 2 | 3 | 4 |
| **Nurse** | Yes・No | times | 1 | 2 | 3 | 4 |  | 1 | 2 | 3 | 4 |
| **Maternity nurse** | Yes・No | times | 1 | 2 | 3 | 4 |  | 1 | 2 | 3 | 4 |
| **Massage practitioner / Acupressure therapist** | Yes・No | times | 1 | 2 | 3 | 4 |  | 1 | 2 | 3 | 4 |
| **Acupuncturist/ Moxibustionist** | Yes・No | times | 1 | 2 | 3 | 4 |  | 1 | 2 | 3 | 4 |
| **Judo therapist (Bonesetter)** | Yes・No | times | 1 | 2 | 3 | 4 |  | 1 | 2 | 3 | 4 |
| **Nutritionist** | Yes・No | times | 1 | 2 | 3 | 4 |  | 1 | 2 | 3 | 4 |
| **Yoga instructor** | Yes・No | times | 1 | 2 | 3 | 4 |  | 1 | 2 | 3 | 4 |
| **Chiropractor** | Yes・No | times | 1 | 2 | 3 | 4 |  | 1 | 2 | 3 | 4 |
| **Manual therapist** | Yes・No | times | 1 | 2 | 3 | 4 |  | 1 | 2 | 3 | 4 |
| **Aromatherapist / Herb therapist** | Yes・No | times | 1 | 2 | 3 | 4 |  | 1 | 2 | 3 | 4 |
| **Spiritual therapist** | Yes・No | times | 1 | 2 | 3 | 4 |  | 1 | 2 | 3 | 4 |
| **Homeopathy therapist** | Yes・No | times | 1 | 2 | 3 | 4 |  | 1 | 2 | 3 | 4 |
| **Other(please specify):** | Yes・No | times | 1 | 2 | 3 | 4 |  | 1 | 2 | 3 | 4 |
| **Other(please specify):** | Yes・No | times | 1 | 2 | 3 | 4 |  | 1 | 2 | 3 | 4 |

**Question ２. Medical care / health services received from physicians**

If you not seen a physician in the past 12 months, please go to question 3.

| Have you received any of the following medical care or health services from physicians **in the last 12 months**? | | Number of times you saw this provider **in the last 3 months**? | Please indicate the **main** reason you **last** received the care / service. (Check only one) | | | | | How helpful was it to receive you care / service from the physician?  (Check only one) | | | |
| --- | --- | --- | --- | --- | --- | --- | --- | --- | --- | --- | --- |
|  |  |  | 1 | 2 | 3 | 4 |  | 1 | 2 | 3 | 4 |
|  |  |  | For an acute illness / condition, one that lasted less than one month | To treat a long-term health condition(one that lasted more than one month) or its symptoms | To improve well-being | Other | (Please specify the other reason) | Very | Somewhat | Not at all | Don’t know |
| **Acupuncture and moxibustion** | Yes・No | times | 1 | 2 | 3 | 4 |  | 1 | 2 | 3 | 4 |
| **Massage** | Yes・No | times | 1 | 2 | 3 | 4 |  | 1 | 2 | 3 | 4 |
| **Dietary supplement** | Yes・No | times | 1 | 2 | 3 | 4 |  | 1 | 2 | 3 | 4 |
| **Cupping** | Yes・No | times | 1 | 2 | 3 | 4 |  | 1 | 2 | 3 | 4 |
| **Aromatherapy** | Yes・No | times | 1 | 2 | 3 | 4 |  | 1 | 2 | 3 | 4 |
| **Herb therapy** | Yes・No | times | 1 | 2 | 3 | 4 |  | 1 | 2 | 3 | 4 |
| **Homeopathy** | Yes・No | times | 1 | 2 | 3 | 4 |  | 1 | 2 | 3 | 4 |
| **Spiritual therapy** | Yes・No | times | 1 | 2 | 3 | 4 |  | 1 | 2 | 3 | 4 |
| **Music therapy** | Yes・No | times | 1 | 2 | 3 | 4 |  | 1 | 2 | 3 | 4 |
| **Spa therapy** | Yes・No | times | 1 | 2 | 3 | 4 |  | 1 | 2 | 3 | 4 |
| **Ayurveda** | Yes・No | times | 1 | 2 | 3 | 4 |  | 1 | 2 | 3 | 4 |
| **(　　　　　　　　)** | Yes・No | times | 1 | 2 | 3 | 4 |  | 1 | 2 | 3 | 4 |
| **(　　　　　　　　)** | Yes・No | times | 1 | 2 | 3 | 4 |  | 1 | 2 | 3 | 4 |
| **(　　　　　　　　)** | Yes・No | times | 1 | 2 | 3 | 4 |  | 1 | 2 | 3 | 4 |

**Question 3. Medical care / Health services received from Korean Medical doctors**

If you not seen a Korean Medical Doctor in the past 12 months, please go to question 4.

| Have you received any of the following medical care or health services from Korean Medical Doctors **in the last 12 months**? | | Number of times you saw this provider **in the last 3 months**? | Please indicate the **main** reason you **last** received the care / service. (Check only one) | | | | | How helpful was it to receive you care / service from the Korean Medical Doctor? (Check only one) | | | |
| --- | --- | --- | --- | --- | --- | --- | --- | --- | --- | --- | --- |
|  |  |  | 1 | 2 | 3 | 4 |  | 1 | 2 | 3 | 4 |
|  |  |  | For an acute illness / condition, one that lasted less than one month | To treat a long-term health condition(one that lasted more than one month) or its symptoms | To improve well-being | Other | (Please specify the other reason) | Very | Somewhat | Not at all | Don’t know |
| **Acupuncture and moxibustion** | Yes・No | times | 1 | 2 | 3 | 4 |  | 1 | 2 | 3 | 4 |
| **Massage** | Yes・No | times | 1 | 2 | 3 | 4 |  | 1 | 2 | 3 | 4 |
| **Dietary supplement** | Yes・No | times | 1 | 2 | 3 | 4 |  | 1 | 2 | 3 | 4 |
| **Cupping** | Yes・No | times | 1 | 2 | 3 | 4 |  | 1 | 2 | 3 | 4 |
| **Aromatherapy** | Yes・No | times | 1 | 2 | 3 | 4 |  | 1 | 2 | 3 | 4 |
| **Herb therapy** | Yes・No | times | 1 | 2 | 3 | 4 |  | 1 | 2 | 3 | 4 |
| **Homeopathy** | Yes・No | times | 1 | 2 | 3 | 4 |  | 1 | 2 | 3 | 4 |
| **Spiritual therapy** | Yes・No | times | 1 | 2 | 3 | 4 |  | 1 | 2 | 3 | 4 |
| **Music therapy** | Yes・No | times | 1 | 2 | 3 | 4 |  | 1 | 2 | 3 | 4 |
| **Spa therapy** | Yes・No | times | 1 | 2 | 3 | 4 |  | 1 | 2 | 3 | 4 |
| **Ayurveda** | Yes・No | times | 1 | 2 | 3 | 4 |  | 1 | 2 | 3 | 4 |
| **(　　　　　　　　)** | Yes・No | times | 1 | 2 | 3 | 4 |  | 1 | 2 | 3 | 4 |
| **(　　　　　　　　)** | Yes・No | times | 1 | 2 | 3 | 4 |  | 1 | 2 | 3 | 4 |
| **(　　　　　　　　)** | Yes・No | times | 1 | 2 | 3 | 4 |  | 1 | 2 | 3 | 4 |
| **(　　　　　　　　)** | Yes・No | times | 1 | 2 | 3 | 4 |  | 1 | 2 | 3 | 4 |

**Question 4. Use of Dietary Supplements(Dietary Supplements, Health Foods, Traditional Korean Medicine, etc.**)

| **《 Vitamins / Minerals 》** Have you used vitamins / minerals **in the last 12 months**? | | | | | | | | | **（　 Yes・No 　）** | | | | |  |
| --- | --- | --- | --- | --- | --- | --- | --- | --- | --- | --- | --- | --- | --- | --- |
| e.g.）Vitamin A, Vitamin B1, Vitamin B2, Vitamin B6, Vitamin B12, Vitamin C, Vitamin D, Vitamin E, Vitamin K, Multiple vitamin, Pantothenic acid,  Biotin, Niacin, Folic acid, Iron, Calcium, Copper, Zinc, Magnesium, Potassium, Multi-mineral etc. | | | | | | | | | | | | | |  |
| Please add the product name used each in the last 12 months.  （Regardless of the physician / Korean medical doctor’s prescription or its advise） | Do you **currently** use the product? | Please indicate the **main** reason that applies to your **last** use. (Check only one) | | | | | | | | How helpful did you find this product?  (Check only one) | | | |  |
|  |  | 1 | 2 | | | 3 | 4 |  | | 1 | 2 | 3 | 4 |  |
|  |  | For an acute illness / condition, one that lasted less than one month | To treat a long-term health condition(one that lasted more than one month) or its symptoms | | | To improve well-being | Other | (Please specify the other reason) | | Very | Somewhat | Not at all | Don’t know |  |
| (　　　　　　　　　　　　) | Yes・No | 1 | 2 | | | 3 | 4 |  | | 1 | 2 | 3 | 4 |  |
| (　　　　　　　　　　　　) | Yes・No | 1 | 2 | | | 3 | 4 |  | | 1 | 2 | 3 | 4 |  |
| (　　　　　　　　　　　　) | Yes・No | 1 | 2 | | | 3 | 4 |  | | 1 | 2 | 3 | 4 |  |
| (　　　　　　　　　　　　) | Yes・No | 1 | 2 | | | 3 | 4 |  | | 1 | 2 | 3 | 4 |  |
| (　　　　　　　　　　　　) | Yes・No | 1 | 2 | | | 3 | 4 |  | | 1 | 2 | 3 | 4 |  |
| (　　　　　　　　　　　　) | Yes・No | 1 | 2 | | | 3 | 4 |  | | 1 | 2 | 3 | 4 |  |
| **《Diet treatment》《Medicinal liquor》** | | | | **（　 Yes・No 　）** | | | | | | | | | |  |
| **Macrobiotic** | Yes・No | 1 | 2 | | | 3 | 4 |  | | 1 | 2 | 3 | 4 |  |
| **Fasting therapy** | Yes・No | 1 | 2 | | | 3 | 4 |  | | 1 | 2 | 3 | 4 |  |
| **Low carbohydrate diet** | Yes・No | 1 | 2 | | | 3 | 4 |  | | 1 | 2 | 3 | 4 |  |
| (　　　　　　　　　　　　) | Yes・No | 1 | 2 | | | 3 | 4 |  | | 1 | 2 | 3 | 4 |  |
| (　　　　　　　　　　　　) | Yes・No | 1 | 2 | | | 3 | 4 |  | | 1 | 2 | 3 | 4 |  |
| (　　　　　　　　　　　　) | Yes・No | 1 | 2 | | | 3 | 4 |  | | 1 | 2 | 3 | 4 |  |
| **Alcohol containing natural ingredients** | Yes・No | 1 | 2 | | | 3 | 4 |  | | 1 | 2 | 3 | 4 |  |
| (　　　　　　　　　　　　) | Yes・No | 1 | 2 | | | 3 | 4 |  | | 1 | 2 | 3 | 4 |  |
| (　　　　　　　　　　　　) | Yes・No | 1 | 2 | | | 3 | 4 |  | | 1 | 2 | 3 | 4 |  |
| **《Homeopathy》** Have you used the homeopathy product **in the last 12 months**?  If you YES, please add the product name and answer questions in below. | | | | | | | | | **（　Yes・No　）** | | | | |  |
| (　　　　　　　　　　　　) | Yes・No | 1 | 2 | | | 3 | 4 |  | | 1 | 2 | 3 | 4 |  |
| (　　　　　　　　　　　　) | Yes・No | 1 | 2 | | | 3 | 4 |  | | 1 | 2 | 3 | 4 |  |
| **《Traditional Korean Medicine》**  Traditional Korean Medicine is classified as follows:  (1) Traditional Korean Medicines (decoction)  (2) Manufactured traditional Korean medicines for prescription  （3) OTC traditional Korean medicines  1. Have you used these Korean Medicines **in the last 12 months**? 2. Do you **currently** use these Korean Medicines?  If you YES, please check in below questions. If you NO, please go to**《 Dietary supplements, Health foods 》**questions. | | | | | | | | | | | | | |  |
| (1) Traditional Korean Medicines (decoction) | | | | | 1. I used it in the last 12 months**（Yes・No）** → 2. Using currently**（Yes・No）** | | | | | | | | |  |
| (2) Manufactured traditional Korean medicines  for prescription | | | | | 1. I used it in the last 12 months**（Yes・No）** → 2. Using currently**（Yes・No）** | | | | | | | | |  |
| (3） OTC traditional Korean medicines | | | | | 1. I used it in the last 12 months**（Yes・No）** → 2. Using currently**（Yes・No）** | | | | | | | | |  |
| Please indicate the Korean Medicine Product you used.  e.g.） Ojeogsan(五積散), Gunghatang(芎夏湯), Ijintang(二陳湯)、 Gumiganghwaltang(九味羌活湯), Pyeongwisan(平胃散), Pyeongwisan(香砂平胃散),  Bojungikgitang(補中益気湯), Socheongryongtang(小青龍湯), Galguentang(葛根湯), Samsoeum(蔘蘇飮)　etc. | | | | | | | | | | | | | |  |
| Please add the name of used Korean medicine. （If you don’t know the product name, describe a disease / symptom when you begin to take the Korean medicine） | Do you **currently** use the product? | Please indicate the **main** reason you **last** used this product. (Check only one) | | | | | | | | How helpful did you find the product?  (Check only one) | | | |  |
|  |  | 1 | 2 | | | 3 | 4 |  | | 1 | 2 | 3 | 4 |  |
|  |  | For an acute illness / condition, one that lasted less than one month | To treat a long-term health condition(one that lasted more than one month) or its symptoms | | | To improve well-being | Other | (Please specify the other reason) | | Very | Somewhat | Not at all | Don’t know |  |
| (　　　　　　　　　　　　) | Yes・No | 1 | 2 | | | 3 | 4 |  | | 1 | 2 | 3 | 4 |  |
| (　　　　　　　　　　　　) | Yes・No | 1 | 2 | | | 3 | 4 |  | | 1 | 2 | 3 | 4 |  |
| (　　　　　　　　　　　　) | Yes・No | 1 | 2 | | | 3 | 4 |  | | 1 | 2 | 3 | 4 |  |
| (　　　　　　　　　　　　) | Yes・No | 1 | 2 | | | 3 | 4 |  | | 1 | 2 | 3 | 4 |  |
| (　　　　　　　　　　　　) | Yes・No | 1 | 2 | | | 3 | 4 |  | | 1 | 2 | 3 | 4 |  |
| (　　　　　　　　　　　　) | Yes・No | 1 | 2 | | | 3 | 4 |  | | 1 | 2 | 3 | 4 |  |
| **《Dietary supplements, Health foods》**  e.g.） Red ginseng, Ginseng, Omega-3 fatty acid, Probiotic, Aloe, Glucosamine, Chondroitin, Saw palmetto, Green juice, Collagen, Placenta, Blueberry etc. | | | | | | | | | | | | | |  |
| **Red ginseng** | Yes・No | 1 | 2 | | | 3 | 4 |  | | 1 | 2 | 3 | 4 |  |
| **Ginseng** | Yes・No | 1 | 2 | | | 3 | 4 |  | | 1 | 2 | 3 | 4 |  |
| **Omega-3 fatty acid** | Yes・No | 1 | 2 | | | 3 | 4 |  | | 1 | 2 | 3 | 4 |  |
| **Probiotic** | Yes・No | 1 | 2 | | | 3 | 4 |  | | 1 | 2 | 3 | 4 |  |
| (　　　　　　　　　　　　) | Yes・No | 1 | 2 | | | 3 | 4 |  | | 1 | 2 | 3 | 4 |  |
| (　　　　　　　　　　　　) | Yes・No | 1 | 2 | | | 3 | 4 |  | | 1 | 2 | 3 | 4 |  |
| (　　　　　　　　　　　　) | Yes・No | 1 | 2 | | | 3 | 4 |  | | 1 | 2 | 3 | 4 |  |
| (　　　　　　　　　　　　) | Yes・No | 1 | 2 | | | 3 | 4 |  | | 1 | 2 | 3 | 4 |  |
| **《Purchase place》** Where did you buy the product?  （Check all that apply） | | 1. Pharmacy 2. Drug store 3. Supermarket 4. Internet shopping 5. Mail order 6. Others（ 　　　） | | | | | | | | | | | |  |

**Question 5. Self-help practices**

This is a last Question. Thank you for your cooperation.

| Have you used any of the following self-help practices **in the last 12 months**?  （Regardless of the physician / Korean medical doctor’s prescription or its advise.） | | Number of times you used practice **in the last 3 months**? | Please indicate the **main** reason that applies to your **last** use of the self-help practice. (Check only one) | | | | | How helpful did you find the self-help practice? (Check only one) | | | |
| --- | --- | --- | --- | --- | --- | --- | --- | --- | --- | --- | --- |
|  |  |  | 1 | 2 | 3 | 4 |  | 1 | 2 | 3 | 4 |
|  |  |  | For an acute illness / condition, one that lasted less than one month | To treat a long-term health condition(one that lasted more than one month) or its symptoms | To improve well-being | Other | (Please specify the other reason) | Very | Somewhat | Not at all | Don’t know |
| **Meditation** | Yes・No | times | 1 | 2 | 3 | 4 |  | 1 | 2 | 3 | 4 |
| **Yoga** | Yes・No | times | 1 | 2 | 3 | 4 |  | 1 | 2 | 3 | 4 |
| **Qigong** | Yes・No | times | 1 | 2 | 3 | 4 |  | 1 | 2 | 3 | 4 |
| **Tai Chi** | Yes・No | times | 1 | 2 | 3 | 4 |  | 1 | 2 | 3 | 4 |
| **Relaxation techniques** | Yes・No | times | 1 | 2 | 3 | 4 |  | 1 | 2 | 3 | 4 |
| **Music therapy** | Yes・No | times | 1 | 2 | 3 | 4 |  | 1 | 2 | 3 | 4 |
| **Picture therapy** | Yes・No | times | 1 | 2 | 3 | 4 |  | 1 | 2 | 3 | 4 |
| **Attend traditional healing ceremony** | Yes・No | times | 1 | 2 | 3 | 4 |  | 1 | 2 | 3 | 4 |
| **Praying for own health** | Yes・No | times | 1 | 2 | 3 | 4 |  | 1 | 2 | 3 | 4 |
| **Electric massage machine** | Yes・No | times | 1 | 2 | 3 | 4 |  | 1 | 2 | 3 | 4 |
| **Other health appliances** | Yes・No | times | 1 | 2 | 3 | 4 |  | 1 | 2 | 3 | 4 |
| **Walking** | Yes・No | times | 1 | 2 | 3 | 4 |  | 1 | 2 | 3 | 4 |
| **Forest therapy** | Yes・No | times | 1 | 2 | 3 | 4 |  | 1 | 2 | 3 | 4 |
| **Aromatherapy** | Yes・No | times | 1 | 2 | 3 | 4 |  | 1 | 2 | 3 | 4 |
| **Hyperthermia** | Yes・No | times | 1 | 2 | 3 | 4 |  | 1 | 2 | 3 | 4 |
| **Magnet therapy** | Yes・No | times | 1 | 2 | 3 | 4 |  | 1 | 2 | 3 | 4 |
| **Spa therapy** | Yes・No | times | 1 | 2 | 3 | 4 |  | 1 | 2 | 3 | 4 |
| **Bath additive** | Yes・No | times | 1 | 2 | 3 | 4 |  | 1 | 2 | 3 | 4 |
| **(　　　　　　　　 )** | Yes・No | times | 1 | 2 | 3 | 4 |  | 1 | 2 | 3 | 4 |
| **(　　　　　　　　 )** | Yes・No | times | 1 | 2 | 3 | 4 |  | 1 | 2 | 3 | 4 |
| **(　　　　　　 　　)** | Yes・No | times | 1 | 2 | 3 | 4 |  | 1 | 2 | 3 | 4 |
